# INVESTIGATING PHYSICAL EXERCISE-RELATED MEDIATING VARIABLES IN THE RDAD-NW TRANSLATIONAL STUDY

**DOI:** 10.1093/geroni/igac059.081

**Published:** 2022-12-20

**Authors:** Jingyi Li, Barbara Cochrane

**Affiliations:** University of Washington, Sammamish, Washington, United States; University of Washington School of Nursing, Seattle, Washington, United States

## Abstract

Declining physical function significantly worsens quality of life in dementia care dyads. Caring for people living with dementia (PLWD) at home is both physically demanding and emotionally stressful. Physical exercise is strongly recommended as a way of sustaining or improving overall function in dementia care dyads. However, little is known regarding the impact of home-based dyadic exercise interventions. We present results from a secondary analysis of data from a multicomponent translational study, Reducing Disability in Alzheimer's Disease – North West (RDAD-NW). We report results from an examination of the mediating role of exercise on the trajectory of change in physical function over time for both PLWD and their care partners.

